# Beyond sex and gender difference in funding and reporting of health research

**DOI:** 10.1186/s41073-018-0050-6

**Published:** 2018-08-28

**Authors:** Olena Hankivsky, Kristen W. Springer, Gemma Hunting

**Affiliations:** 1 School of Public Policy, Institute for Intersectionality Research and Policy, Simon Fraser University, Harbour Centre Campus, Room 3274, 505 West Hastings Street, Vancouver, BC V68 5K3 Canada; 2 Department of Sociology, Faculty Affiliate, Institute for Health, Health Care Policy and Aging Research, Rutgers, The State University of New Jersey, 26 Nichol Avenue, New Brunswick, NJ 08901 USA; 3 Institute for Intersectionality Research and Policy, Simon Fraser University, Harbour Centre Campus, Room 3274, 505 West Hastings Street, Vancouver, BC V68 5K3 Canada

**Keywords:** Sex differences, Sex/gender, Gender, Health, Knowledge generation, Intersectionality, Policy, Funding agencies, Scientific reporting, Health journals

## Abstract

**Background:**

Understanding sex and gender in health research can improve the quality of scholarship and enhance health outcomes. Funding agencies and academic journals are two key gatekeepers of knowledge production and dissemination, including whether and how sex/gender is incorporated into health research. Though attention has been paid to key issues and practices in accounting for sex/gender in health funding agencies and academic journals, to date, there has been no systematic analysis documenting whether and how agencies and journals require attention to sex/gender, what conceptual explanations and practical guidance are given for such inclusion, and whether existing practices reflect the reality that sex/gender cannot be separated from other axes of inequality.

**Methods:**

Our research systematically examines official statements about sex/gender inclusion from 45 national-level funding agencies that fund health research across 36 countries (covering the regions of the EU and associated countries, North America, and Australia) and from ten top-ranking general health (the top five in “science” and the top five in “social science”) and ten sex- and/or gender-related health journals. We explore the extent to which agencies and journals require inclusion of sex/gender considerations and to what extent existing strategies reflect state of the art understandings of sex/gender, including intersectional perspectives.

**Results:**

The research highlights the following: (a) there is no consistency in whether sex/gender are mentioned in funding and publishing guidelines; (b) there is wide variation in how sex/gender are conceptualized and how researchers are asked to address the inclusion/exclusion of sex/gender in research; (c) funding agencies tend to prioritize male/female equality in research teams and funding outcomes over considerations of sex/gender in research content and knowledge production; and (d) with very few exceptions, agency and journal criteria fail to recognize the complexity of sex/gender, including the intersection of sex/gender with other key factors that shape health.

**Conclusions:**

The conceptualization and integration of sex/gender needs to better capture the interacting and complex factors that shape health—an imperative that can be informed by an intersectional approach. This can strengthen current efforts to advance scientific excellence in the production and reporting of research. We provide recommendations and supporting questions to strengthen consideration of sex/gender in policies and practices of health journals and funding agencies.

## Background

Scholars, policy makers, and healthcare providers worldwide have argued that improving the quality and rigor of scientific evidence requires taking into account sex and gender as key factors in health research [1–8]. The inclusion of sex/gender^1^ is considered essential for addressing knowledge gaps and producing more accurate and comprehensive information about gendered health experiences, interactions with the health care system, unequal/unfair health outcomes, and the meaning (and measurement) of health itself.

Most recently, significant attention has shifted to funding agencies and scientific journals and how they can be harnessed to require researchers to address sex/gender in their research [7–19]. To date, there have been a number of attempts to highlight promising practices of national-level funding agency policies (e.g., [5, 11, 13, 20]), to report on North American and EU national-level funding agency trends in relation to sex/gender (e.g., [21, 22]), and to review and respond to existing sex/gender policies within academic journals [7, 23]. However, we know of no systematic analysis documenting to what extent both national health funding agencies and scholarly journals require attention to sex/gender, what conceptual explanations and practical guidance are given for such inclusion, and finally, whether existing practices reflect the reality that sex/gender cannot be separated from other axes of inequality.

Our research addresses this gap by systematically examining official statements about sex/gender inclusion from 45 national funding agencies that fund health research across 36 countries (covering the regions of the EU and associated countries, North America, and Australia) and from 10 top-ranking general health and 10 sex- and/or gender-related health journals. The purpose of the study is two-fold: to determine the extent to which key agencies and select journals require any inclusion of sex/gender considerations and to what extent existing strategies reflect state of the art understandings of sex/gender, including how sex/gender interact with a myriad of other factors (e.g., race, ethnicity, socio-economic status, age) to shape health. Investigating funding organizations and journals in tandem is logical because they are inextricable. As Del Boca explains, “sex/gender issues in the conduct of scientific studies are mirrored in the scholarly journals that publish that research” (p.1) [9]. At the same time, we place special emphasis on funding agencies as they are instrumental in generating research—knowledge production—which then in turn, scholarly journals report. Based on our findings, we provide recommendations and supporting questions that journals, agencies, and scholars can use to ensure scholarship is more consistent with contemporary understandings of sex/gender. Specifically, our intervention is aimed at improving the conceptualization and application of sex/gender to better capture the plethora of interacting and complex factors that shape health and which can inform the efforts of funding agencies and journals to advance scientific excellence in the production and reporting of health research.

### Contemporary framings of sex/gender

While the importance of understanding biological and social factors associated with the health of men and women is widely acknowledged, conceptual and methodological approaches for actually doing this have evolved significantly. Understandings have deepened about the complex relationship between sex/gender [24], and new insights have emerged about how sex/gender interfaces with other determinants of health including socioeconomic status, race, ethnicity, sexuality, and geographic location. In using the term sex/gender we are arguing that there are very few cases of stand-alone “sex biology” and that discussion and analyses of “sex” should proceed with the assumption of sex/gender interconnectedness unless proven otherwise – despite the reality that “sex” and “gender” are often separated in research and policy application.^2^ One can imagine the concept of gender without links to specific biological factors, and we therefore discuss gender, not sex/gender, when discussing social factors not linked to biology. We also highlight, as elaborated on below, that discussions of social factors in the field have extended beyond static and stand-alone considerations of gender [25].

For example, in a 2012 special issue of Gender and Health for *Social Science and Medicine,* Springer et al. [26] outline two cutting edge strategies for understanding sex/gender in the context of health research: relational and intersectional approaches. Relational constructions of gender recognize gender as dynamic and situational, and prioritize attention to differences among women and men, and understand gender as a property of social norms, relationships, structures, ideologies, etc. rather than something a person embodies. For example, understanding gender in this way could include exploring the gendered health effects of family leave policies or understanding the consequences of the male breadwinner norm. Understanding gender as structural/relational also means that gender is so deeply embedded in social life that it is rarely (if ever) possible to separate sex from gender in human beings, and therefore sex/gender should be modeled and explored together as the default. When the intersection of sex/gender cannot be empirically investigated, it is essential that the intersection be theorized and used as a lens to articulate any “sex” effects.

One incredible benefit of acknowledging and modeling “sex” as integrally intertwined with gender is the necessary conclusion that any male/female difference is not a “sex” (read biological) difference. Indeed, rigorous sex/gender analyses require taking the biological, as well as social, structural aspects of male/female health differences very seriously. As such it is imperative to understand that sex is not a mechanism. If the proposed mechanism male/female health differences are (in part) biological differences—then model those biological differences. This is good science. For example, if the proposed male/female health difference results from body fat differences, then study body fat differences and not “sex.” Be specific about the biological mechanisms and study those—or at the very least theorize and articulate those mechanisms if they cannot be directly studied (see reference [25] for further explication and more examples).

Intersectionality prioritizes the interaction of various factors and structures in the construction of health, such as sex/gender, age, race/ethnicity, and socioeconomic status, and in so doing, decenters the prioritization of sex/gender in health research and policy analysis to allow for more nuanced, diversity sensitive, and complete understandings of the plethora of health determinants. Intersectionality also goes beyond a simple “additive” approach (e.g., that is sex/gender plus attention to other factors). Instead, the perspective advances an understanding of how various factors, including but not limited to sex/gender, relate and interact with one another at a group, process, and structural level [27–30]. Intersectionality can be applied to understanding individual level experiences but always in the context of attention to broader social divisions. Focusing on a broad set of interacting factors produces evidence that more accurately captures the complexity and diversity of health [2, 27, 31]. Accordingly, intersectionality is now well established as a key and leading framework for accurately understanding and responding to health inequities and for improving health [29, 31–43].

In sum, relational and intersectional approaches reflect the most recent theoretical developments in the field and have the potential to disrupt a number of problematic trends in sex/gender research including: binary constructions of sex (male vs. female) and gender (masculine vs. feminine), the treatment of sex and gender as easily separable, and the disconnection of sex/gender from other health-influencing factors. As the research articles in the Springer et al. [26] special issue persuasively illustrate, relational and intersectional approaches capture sex/gender complexities and highlight the need to capture intersections of biological factors and other forms of social differences—including but not limited to sex/gender. In sum, these approaches can better and more accurately illuminate the diverse health of men and women, and in so doing, produce better science and ultimately improved health outcomes.

### Existing guidance for integrating sex/gender in health research and journal reporting

Funding agencies and journals have been referred to as “change agents” [20] because they have the potential to improve the accuracy and rigor of knowledge production and reporting on current and emerging health challenges across populations. Not surprisingly, there has been increased attention to how improvements can be made in the way sex/gender is approached in research, reporting, and the peer review process. For example, in Table 1 below, Gahagan et al. [7] have produced instructions intended to support researchers and peer reviewers by directing them toward relying on important tools and resources.

**Table 1:** Instructions for authors and peer reviewers could include

1. Examples of sex and gender definitions on journal websites to ensure accuracy; 2. Resources for authors about best practices on sex and gender analysis in their research field; 3. Online resources for training of new peer reviewers on the roles of sex and gender in both basic science and health research; and 4. Links to existing training materials for health researchers and peer reviewers that have been, or are being developed, by organizations such as CIHR, NIH, GenderNet, and others. [7]	

More recently, Heidari et al. published SAGER (Sex and Gender Equity in Research) guidelines (Table 2 directly below) that include general principles and prompts to help standardize sex and gender reporting in scientific publications [23].

**Table 2:** Sex and Gender Equity in Research (SAGER) guidelines: general principles

• Authors should use the terms *sex* and *gender* carefully in order to avoid confusing both terms. • Where the subjects of research comprise organisms capable of differentiation by sex, the research should be designed and conducted in a way that can reveal sex-related differences in the results, even if these were not initially expected. • Where subjects can also be differentiated by gender (shaped by social and cultural circumstances), the research should be conducted similarly at this additional level of distinction. [23]	

These guidelines are intended to be applicable to all research with humans, animals, or any material originating from humans and animals (e.g., organs, cells, tissues). Also included in the SAGER guidelines is an authors’ checklist for gender-sensitive reporting (Table 3 below).

**Table 3:** Authors’ checklist for gender-sensitive reporting

Research approaches	
✓ Are the concepts of gender and/or sex used in your research project?	
✓ If yes, have you explicitly defined the concepts of gender and/or sex? Is it clear what aspects of gender and/or sex are being examined in your study?	
✓ If no, do you consider this to be a significant limitation? Given existing knowledge in the relevant literature, are there plausible gender and/or sex factors that should have been considered? If you consider sex and/or gender to be highly relevant to your proposed research, the research design should reflect this.	
Research questions and hypotheses	
✓ Does your research question(s) or hypothesis/es make reference to gender and/or sex, or relevant groups or phenomena (e.g., differences between males and females, differences among women, seeking to understand a gendered phenomenon such as masculinity)?	
Literature review	
✓ Does your literature review cite prior studies that support the existence (or lack) of significant differences between women and men, boys and girls, or males and females?	
✓ Does your literature review point to the extent to which past research has taken gender or sex into account?	
Research methods	
✓ Is your sample appropriate to capture gender and/or sex-based factors?	
✓ Is it possible to collect data that are disaggregated by sex and/or gender?	
✓ Are the inclusion and exclusion criteria well justified with respect to sex and/or gender? (Note: this pertains to human and animal subjects and biological systems that are not whole organisms)	
✓ Is the data collection method proposed in your study appropriate for investigation of sex and/or gender?	
✓ Is your analytic approach appropriate and rigorous enough to capture gender and/or sex-based factors?	
Ethics	
✓ Does your study design account for the relevant ethical issues that might have particular significance with respect to gender and/or sex? (e.g., inclusion of pregnant women in clinical trials)	

Source: Adapted from Canadian Institutes of Health Research (2016) [53]

The first problem in directing researchers to existing approaches, resources, and training materials is that they simply replicate the status quo. Second, the general guidelines proposed by Heidari et al. [23] emphasize the importance of proper conceptualization of sex/gender but provide definitions in the appendix that place researchers at risk for replicating approaches that fail to capture the fundamentally interconnected relationship of sex/gender. And, in the more detailed instructions to authors regarding “recommendations per section of the article,” a statement is made that “authors should consider all possible explanations for sex- and gender-related phenomena including social, cultural, biological and situational factors, recognizing that many sex-related behaviours might result from either cultural factors or biological factors” (p.4) [23] and in doing so, subsumes the importance of other factors to sex/gender. While these efforts are an important start, they do not sufficiently incorporate contemporary conceptualizations of sex/gender and provide little guidance on how to situate sex/gender in relation to other factors that shape and influence health.

## Methods

**Fig. 1:**
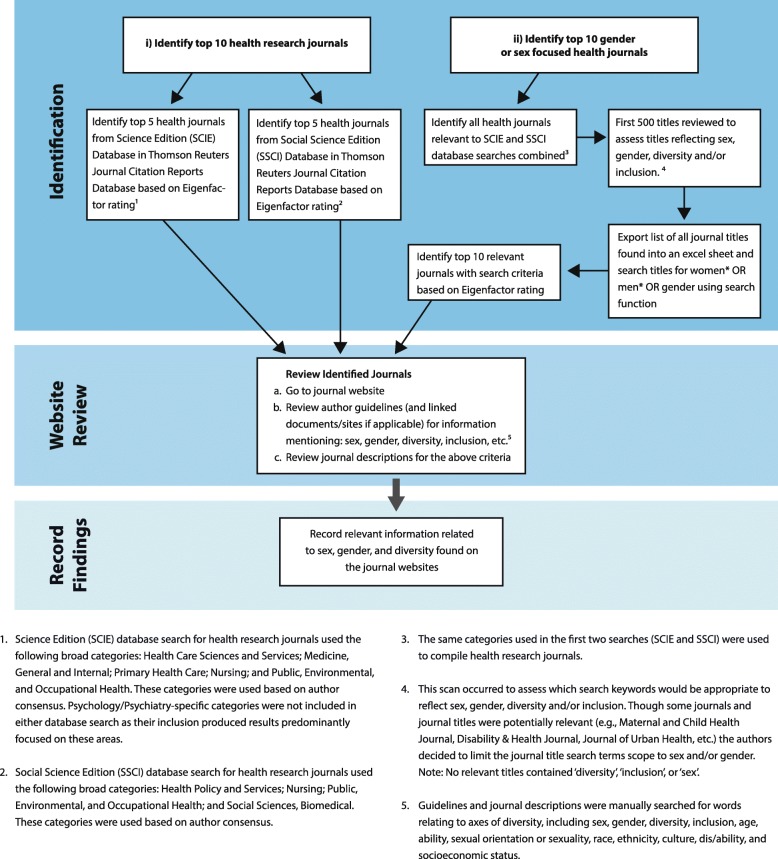
Health journal search

**Fig. 2:**
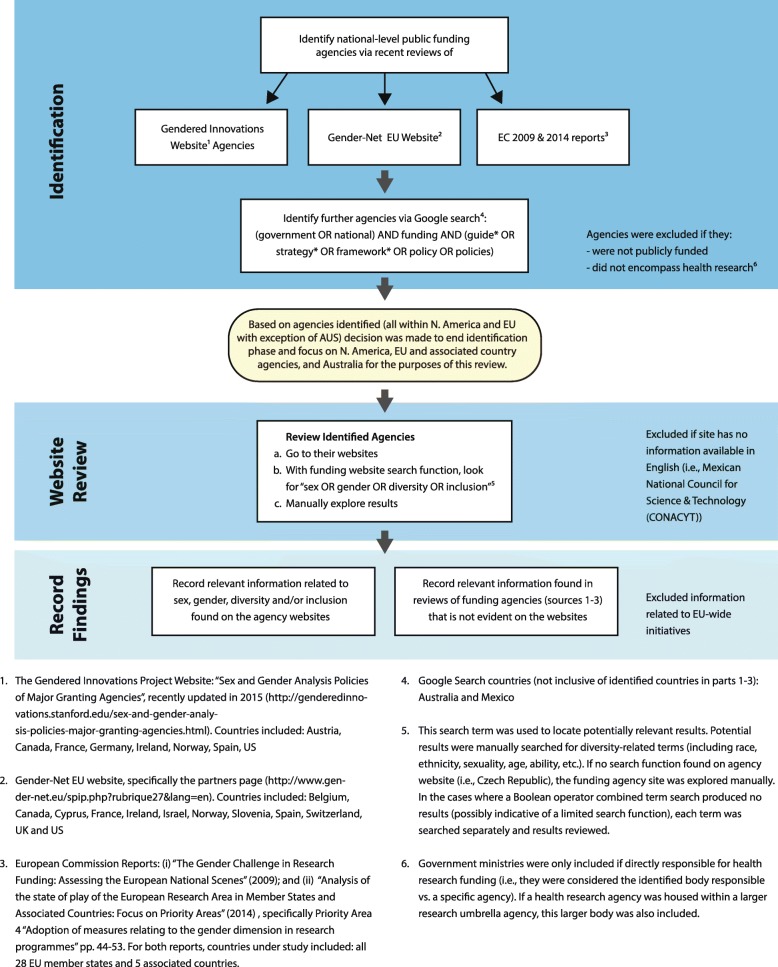
Funding agency search

Our research focused on two distinct areas of research inquiry: peer-reviewed scientific journals and national-level public funding agencies (See Figs. 1 and 2 for details of the search strategies).

For our search of peer-reviewed scientific journals (see Fig. 1), we focused on two overarching sets of journals—health-research journals more generally and health-research journals focused on gender and/or sex more specifically. Journals were located using Thomson Reuters Journal Citation Reports database. To identify the top ten health-research journals, we chose the top five journals in the Science Edition (SCIE) database for 2014, and the top five journals in the Social Science Edition (SSCI) database for 2014.^3^ Journal ranking was based on Eigenfactor ratings. To identify the top ten gender and/or sex focused journals, top health journals relevant to both databases combined that contained (women* OR men* OR gender) in the title were examined.^4^ For all journals, the author guidelines on each journal website (and any other documents that were linked to guideline information) were reviewed. This review involved a search for information mentioning sex, gender, diversity, inclusion, and/or factors related to diversity (e.g., race, ethnicity, age, etc.).

Our search of key funding agencies (see Fig. 2) included a review of existing multi-country inventories including (1) the “Gendered Innovations Project” website [11], which contains information related to “major granting agencies” from across the USA, EU member states, and Canada on existing methods for sex and gender analysis; (2) The Gender-Net EU website [44], a European Research Area Network (ERA-NET) composed of 13 national programme owners from the EU and associated countries and North America working to promote gender equality through structural change in research institutions and integrating sex and gender into research analyses; (3) two key European Commission reports: “The Gender Challenge in Research Funding: Assessing the European National Scenes” [45], and “Analysis of the state of play of the European Research Area in Member States and Associated Countries: focus on priority areas” [22]; and (4) a Google search for national-level public funding bodies that may have been missed in the aforementioned inventories.^5^ Funding agencies differed in their range and scope.

We used the framework of “Gendered Innovations Project” as the foundational template for our assessment of funding organizations, and then expanded on this excellent foundation by systematically searching each identified agency’s website for content relevant to sex, gender, diversity, and/or inclusion (including race, ethnicity, sexuality, age, ability, etc.).

## Results

### Research journals

The top ten health research journals demonstrated differences between the social science and science groupings. Among the top five journals in the Social Science Edition Category, only the *American Journal of Public Health* contains a directive about using “non-discriminatory language” (which includes sexist language). In terms of sex/gender or attention to diversity, the only direction provided to authors is the following instruction from the *American Journal of Public Health* author guidelines: “[i]f race/ethnicity is reported, the authors should indicate in the methods section why race/ethnicity was assessed, how individuals were classified, what the classifications were, and whether the investigators or the participants selected the classifications” (p.22) [46].

Among the top five journals in the Science Edition Category, the situation is somewhat different. Except for the *New England Journal of Medicine*, all of the journals include some directions to authors about addressing sex/gender in reporting, including a section on “reporting sex” in the *Journal of the American Medical Association* (*JAMA*). There is also attention to factors in addition to sex/gender, most notably race/ethnicity (*Lancet* and *JAMA*) and also age (the *British Medical Journal*), and in the case of the Cochrane Database of Systematic Reviews, explicit attention to the factors that contribute to disadvantage including residence, race/ethnicity, occupation, sex/gender, religion, education, socio-economic position, and social capital. This is in line with the International Committee of Medical Journal Editors [47], which has explicitly recognized the importance of age, race, and ethnicity (in addition to sex/gender) for the conduct, reporting, editing, and publication of scholarly work in medical journals.

In terms of the top ten sex and/or gender focused journals that also discuss health, none have explicit reporting guidelines regarding sex/gender or diversity.

### National Research Funding Organizations

**Fig. 3:**
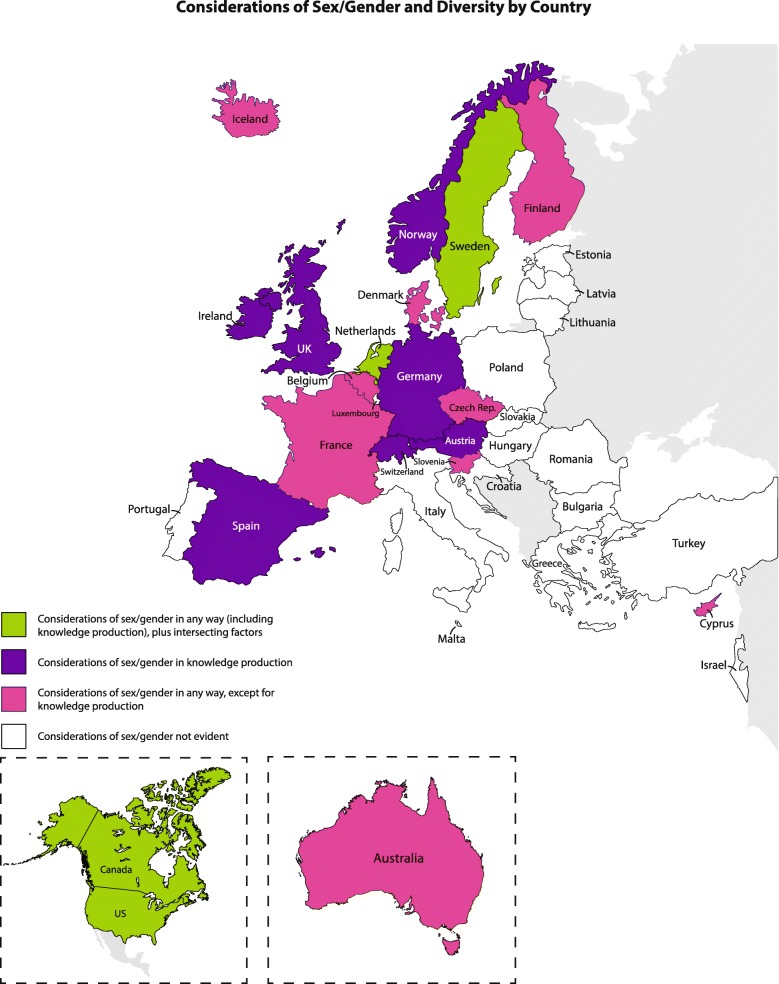
Considerations of sex/gender and diversity by country

Across 36 countries, 20 countries had at least one funding agency that included some discussion of sex/gender on their website and/or secondary sources (see Fig. 3 for map reflecting considerations of sex/gender and diversity by country and Fig. 2 for search methods). This accounted for 28 out of 45 funding agencies.

The majority of these agencies focus on how to improve the underrepresentation of women in scientific research, including creating mechanisms for advancing male/female parity in research teams, organizational structures, and funding success outcomes. For example, the Austrian Science Fund (FWF) [48] reports that “Since 2010, therefore, the FWF has prescribed a target quota of 30% female principal investigators/faculty members, and applicants are required to provide reasons in cases where this target level is not reached.”

In comparison, less attention is paid to the actual sex/gender-related content of research in funding applications, specifically knowledge production*.* For example, only 15 agencies (in Austria, Canada, Germany, Ireland, the Netherlands, Norway, Spain, Sweden, Switzerland, United Kingdom, and United States) recognize the importance of sex/gender in *research content*.

Further, only six funding agencies (in Canada, the Netherlands, Sweden, and the US) specifically pay attention to factors of health beyond sex/gender in a way that would be consistent with an approach that responds to intersectionality—at least for some of their research programs.

## Discussion

Our research findings, summarized above, show that there is no consistency in whether sex/gender is even mentioned in funding and publication guidelines, and this is even the case with scientific journals that are specifically focused on sex/gender. Our data also reveal that requirements that have been institutionalized within funding agencies tend to prioritize greater male/female equality in research teams and funding outcomes over considerations of sex/gender in research *content* and *knowledge production.* As we detail below, within these areas, there is wide variation in how sex/gender are conceptualized and how researchers are asked to address the inclusion/exclusion of sex/gender in their research. Further, with very few exceptions, guidelines and criteria in both funding agencies and journals fail to recognize the real complexity of sex/gender, including the intersection of sex/gender with other key factors that shape health.

### Knowledge production in national funding agencies: sex/gender treatment and limitations

One key finding in our research is that even when sex/gender knowledge production is addressed by national funding agencies, this does not mean that the agencies are modeling best practices for how sex/gender should be conceptualized and operationalized to best generate information and evidence to inform policy and practice.

For example, in some agencies such as the NIH, the focus on sex as a biological variable is emphasized. To illustrate, starting in 2016, grant applicants are asked to “explain how relevant biological variables, such as sex, are factored in to research designs and analyses for studies in vertebrate animals and humans” [49, 50]. Although NIH acknowledges and encourages attention to gender issues in health, as well as biological factors, the focus is clearly on sex as a biological variable. Such an approach runs contrary to contemporary understandings of sex/gender and health that emphasize the fact that it is not possible to identify a pure “sex” (i.e., physiologic) effect that is not influenced by “gender” (social and structural factors) [25]. However, it may be possible to have gender not shaped by sex, as articulated earlier. Therefore, it is inaccurate and misleading to routinely and unquestionably report “sex” effects or “sex” differences as if to imply they operate independently of social construction.

Second, other agencies conflate gender and sex, rather than see them as intertwined. For example, although the Research Council of Norway states that gender is a mandatory criterion in the assessment of grant applications, it provides the following definition which subsumes sex into gender: “Gender as a perspective implies that biological and social gender is reflected in research content. A growing number of studies show that diversity, including gender balance and gender perspectives, helps to enhance the scientific quality and social relevance of research” (p.4) [51]. Moreover, while it directs research to ensure that men and women must be represented in the groups being studied, the council directs researchers to consider “whether the significance of the research results will be different for women and men,” (p.10) [51] minimizing the important possibility that similarities could also be discovered. Statements, guides, and toolkits that advance this difference framing lack nuance and risk essentializing difference. Sex/gender may have relevance for an array of outcomes but such statements may lead researchers to miss important similarities between men and women while also leading to high rates of statistically false “positives” for difference [25, 28, 52].

Among those agencies that pay attention to sex/gender, with very few exceptions (e.g., Canadian Institutes of Health Research (CIHR), Irish Research Council (IRC), Austrian Science Fund (FWF), US National Institutes of Health (NIH)), there is little guidance provided for “how to” conceptualize or actually integrate sex/gender in their work. Agencies often state the importance of a “gender perspective,” “gender dimension,” “social differences between men and women in health research,” “biological sex,” or “sex/gender” without actually demonstrating what this would entail in a research application or how it would transform the design of a research application.

Within the agencies that actually focus on sex/gender in knowledge production, the dominant approach is to distinguish between sex and gender—as if separable, such as those found in CIHR and *Toolkit: Gender in EU-funded research* used by the Irish Research Council and Austrian FWF. For example, since 2010, the CIHR (which houses the only specific funding institute dedicated to gender, sex, and health research in the world—the Institute of Gender and Health (IGH)) has also distinguished itself internationally by implementing a mandatory requirement that all applicants indicate whether and how they are taking sex/gender into account in their research by answering the following questions:

- Are sex (biological) considerations taken into account in this study? Yes/No
- Are gender (socio-cultural) considerations taken into account in this study? Yes/No
- If YES please describe how sex and/or gender considerations will be considered in your research design.
- If NO please explain why sex and/or gender considerations are not applicable in your research design.

CIHR applicants can refer to the *Gender, Sex, and Health Research Guide: A Tool for CIHR Applicants* [53] to assist in answering these questions. The tool is divided into distinct steps in the research process: research questions and hypotheses, literature review, research questions, research methods, and ethics. The tool primarily focuses on sex and/or gender but does direct researchers to consider differences within men and women in their potential research questions.

Further, the Irish Research Council “requires all applicants to indicate whether a potential sex and/or gender dimension may be present or could arise in the course of their proposed research: and, if so, outline how sex/gender analysis will be integrated in the design, implementation, evaluation, interpretation and dissemination of the results” [54]. The council directs researchers to fill out a sex-gender dimension statement, and the Austrian FWF explains how to account for gender in all phases of the research cycle by referring to the *Toolkit: Gender in EU-funded research* [21], which includes similar questions to those found in the CIHR Research Guide. The FWF divides its questions for taking gender into account in research content into categories of research ideas phase, proposal phase, research phase, and dissemination phase.

Some offices of the NIH—such as the NIH Office of Women’s Health—provide useful guidance including an infographic defining sex and gender, along with providing examples of how both sex and gender affect particular health conditions. This is an important and encouraging start, especially as the graph acknowledges that “While sex and gender are distinct concepts, their influence is often inextricably linked” [55].

NIH has also produced recommendations for incorporating sex/gender in health research, emphasizing the necessity of reporting “One overarching feature of considering sex (and gender) in biomedical research is the essentiality of reporting at every stage” (p.2) [56].

### Sex/gender interactions with other axes of inequality

In terms of journals, as was noted in the findings above, there is some acknowledgment of factors beyond sex/gender, but these are predominantly limited to age and race/ethnicity, thus excluding consideration of a more comprehensive possibility of health affecting influences. Moreover, none of the journals we examined acknowledge complex relations and interactions between factors, in a manner that would be considered consistent with an intersectional approach. Instead, different factors are treated in more additive, grocery list fashion.

When sex/gender are specifically addressed in funding agencies, there is a tendency to present sex/gender as primary and dominant influences on all domains of health. Importantly, these approaches fail to properly contextualize the interactions of sex/gender with other axes of inequality and can therefore fail to advance understandings of critically important differences among women and men.

Exceptions include the Swedish Research Council, which explicitly states that it strives “to take into account how categories other than gender can also lead to an evaluation bias or create status hierarchies that interact with the gender power structure” (p.8) [57]. Another important exception is the Netherlands Organization for Health Research and Development (ZonMw) which exists under the Netherlands Organization for Scientific Research (NWO). According to the agency:

attention to diversity and target group differentiation by characteristics such as sex, age, socio-economic situation, educational level, migratory and cultural backgrounds, and sexual inclination, inasmuch as these are relevant to the theme of the project. [58]

At the same time ZonMw does note that “We are currently looking to develop more specific guidelines for assessment of grant applications in terms of considerations of diversity, as well as checklists to help researchers better integrate gender and other forms of diversity in their research” (personal correspondence July 2016). The final European example is that of the UK Research Council which probes applicants to consider the following: Which individuals or groups are likely to be affected by this policy/project/initiative? What is the likely impact on these groups, and how have you arrived at this judgement? If there is potential for a negative impact, what actions can be taken to mitigate the effect? Can this policy/project/initiative be used to help promote equality and diversity? And, importantly, the council encourages public participation in relevant research projects to ensure that there is an opportunity for a wide range of voices involved in the research.

There are also indications that within some quarters of the NIH and CIHR in North America, important shifts are occurring—albeit slowly. For example, the NIH requires reporting sex/gender, race, and ethnicity inclusion information for clinical research (as required by the NIH Policy on the Inclusion of Women and Minorities in Clinical Research), and the format does ask scholars to report sex/gender and race/ethnicity together, showing progress toward being able to identify groups of people with multiple marginalized identifiers. However, only applications for applicable phase III clinical trials must go beyond reporting inclusions and must include a “description of plans to conduct valid analyses” of sex/gender and/or race/ethnic differences [59].

In 2015 the CIHR and SSHRC (Social Sciences and Humanities Research Council) in Canada developed guidelines for integrating sex/gender (specifically called Healthy and Productive Work, SPOR Networks in Chronic Disease) in which some outcomes for diverse patient population sub-groups is emphasized and social determinants (ethnicity, income, occupation) are recognized [60]. Problematically, however, these are reduced to social determinants *of gender*, rather than important factors in their own regard. The CIHR IGH has also introduced online training modules [61]. While they focus on sex and/or gender, there are a few places that additional factors are highlighted. For example, in the module “Sex and Gender in Primary Data Collection with Humans”, an important point is made that measuring and profiling participants on variables that interact with sex or gender will lead to better understanding of what works for whom and under which circumstances [62]. The module on “Sex and Gender in Biomedical Research” trains scholars about why it is important to think about biologically based “sex” characteristics in health research, with a focus on training application reviewers. While nominally about sex/gender, the attention to gender is almost non-existent with the exception of defining the terms of sex and gender [62].

While such developments are promising, they are not systematic across funding agencies nor are they always consistently applied within agencies. Moreover, few agencies have actually developed specific guidance on how to operationalize an approach that properly contextualizes sex/gender within an intersectionality framework.

### Recommended paradigmatic shifts for funding agencies and scientific journals

Taking into account the research findings and shortcomings discussed above, we propose an alternative, more comprehensive and arguably more accurate description of criteria and concomitant lines of interrogation that can be used by researchers, funding agencies, and peer-reviewed journals. In proposing such guidance, we draw on the inclusive approach of the NIH, the diversity-focused approach of ZonMw, recommendations of the International Committee of Medical Journal Editors [47], and critical insights from academic literature (e.g., [26, 29, 63]) that clarify sex/gender and their interactions with other social locations and equity variables in health research contexts. A short time ago, Gahagan raised the need for such focus, concluding that the importance of considering the overlapping and intersecting nature of other key modifiable determinants of health (e.g., income, housing) and non-modifiable determinants of health (e.g., genetics, race) has been less well recognized, integrated, and formalized into research funding processes and publication policies (p.140e) [7].

As laid out in Table 4, we believe there are two central paradigmatic shifts, operationalized by six questions, which if adopted by journals and funding agencies, would fundamentally improve the quality of sex/gender and health research.

**Table 4:** Recommendations for sex/gender and health research

Two paradigmatic shifts needed to fundamentally improve the quality of sex/gender and health research:	
1. Sex/gender should not only be recognized, but also understood as intersecting with other axes of inequality such as race, ability, socioeconomic status, geographic location, sexual orientation, and age.	
2. Gender should be conceptualized as a structural/social determinant of dealth, and should accompany any investigation of “sex” differences—in other words, research should not assume or proceed with the idea that “sex” can be separated from gender.	
Six questions to help operationalize these paradigmatic shifts:	
1. Does the study automatically give primacy to sex/gender? Does it move beyond asking whether sex/gender considerations are taken into account to explaining what relevant factors are taken into account to understand a particular illness, disease or health experience?	
2. How does the study (biomedical, clinical, health systems, or population health focused) identify relevant factors that shape and determine health (e.g., ethnicity/race, sex/gender, age, socio-economic status, geographic location, sexual orientation)? What are the inclusion/exclusion criteriain relation to this question?	
3. How does the research design (data collection and analysis) capture the relationships and interactions (e.g., using a multi-level analysis linking individual experiences to broader social structures) among pertinent health determinants and factors, including, but not limited to, sex/gender? Is the sample size adequate for capturing diversity between and within groups often treated in homogeneous manner (e.g., women, men)?	
4. Does the study conceptualize and/or model gender as a social/structural determinant of health? a. If yes, how? b. If no, has a strong rationale been provided for ho*w*/why a gender conceptualization is not needed—even if the researcher was not able to directly test the gender mechanism?	
5. Does the study assert male/female differences in health related to biological mechanisms? a. If yes, how are those biological mechanisms specifically explained and/or tested? Also, has it been explicitly described how gender and other intersecting factors are intertwined with these biological mechanisms? b. If no, does the study specifically state/demonstrate that intersecting social processes can cause the same biological mechanisms leading to male/female differences in health?	
6. Where relevant, does the study contextualize research findings undertaken with human subjects within broader social structures and processes of power?	

Using these questions as guidance, we improve upon what currently exists within funding agencies and journals, and moreover, extend beyond principles, guidelines, and checklists that we detailed above and are considered best and promising practices in the field. Most importantly, these alternatives raise important considerations that are at the heart of understanding the complexities, interactions, and processes among different factors, structures, and processes inherent in how health inequities are created and sustained.

### Limitations

There are a number of limitations in this study. First, categories chosen to pinpoint health journals were broad in scope, but may have excluded potentially relevant journals; sex and gender health journals were chosen based on title (see Fig. 1 for exclusion criteria details). Second, the research captured national-level government funded/public research funding agencies in North America, EU/associated countries, and Australia (see Fig. 2 for exclusion criteria details), excluding those outside these geographic regions. Agencies analyzed were limited to these regions as no relevant agencies beyond these regions were found via our search methodology, including recent agency reviews and an online search (see Fig. 2). Agencies were also excluded if information was not available in English. Further, our analysis was limited to information available within existing reports and websites; we did not investigate specific contexts to understand general trends that may be affecting the priorities of national research agencies nor did we conduct interviews with agency staff or editors of journals. These would be important future steps to deeper understandings of the interworking, complex, and political nature of funding agencies and journals. Nevertheless, the findings capture important trends in relation to sex/gender and reveal significant limitations in how both key journals and funding agencies conceptualize and guide the production and dissemination of research.

## Conclusion

The findings of our study demonstrate that both funding agencies and scientific journals are not fulfilling their potential as change agents in terms of reflecting and advancing the most accurate and contemporary understandings of sex/gender, including how sex/gender interact with a myriad of other factors.

Health research and reporting needs to better capture interlocking inequities and dimensions of difference including socio-economic status, race/ethnicity, sexual orientation, and geographic location, and in the process, decenter sex and gender as the preferred axes through which to frame all research. This does not mean that sex/and or gender cease to be relevant or significant, but rather that they should be interpreted within a more comprehensive framework, informed by intersectionality and prioritizing diversity.

The paradigmatic shift and guiding questions we propose in this article are intended to contribute to this change. Such changes will need to be supported by further education, training, resources, and supports – similar to initial efforts that have brought attention to the importance of sex/gender in health research and reporting. However, aligning what is already known to be the state of the art knowledge to accurately position sex/gender is essential for continuing the process of improving scientific quality and rigor, improving efficiencies, reducing potential harm due to incomplete evidence, and ultimately improving health for all.

## Abbreviations

CIHR: Canadian Institutes of Health Research
FWF: Austrian Science Fund
IGH: Institute of Gender and Health (CIHR)
JAMA: Journal of the American Medical Association
NIH: US National Institutes of Health
SAGER: Sex and Gender Equity in Research guidelines
SCIE: Science Edition database
SSCI: Social Science Edition database
ZonMw: Netherlands Organization for Health Research and Development

## Footnotes

1 In this article, we use the term sex/gender throughout to represent the inextricable interconnected nature of biology (usually thought of as “sex”) and social environment/structure (usually thought of as “gender”). Importantly, we understand gender not at something people are, but rather as part of norms, interactions, and behaviors, as well as embedded in institutions, policies, and organizations. We refer to “sex” or “gender” or “sex and/or gender” to reference issues when they are used separately and when others specifically use these terms independently

2 Male/female birth weight difference in Jordan is one example of seemingly “pure” sex-based differences that is very much affected by gender. Specifically, girl babies in Jordan (a country with a strong male child preference) have lower birth weight than boy babies, but this difference is in part due to the fact that mothers who know they are carrying a female fetus seek significantly less prenatal care than women who know they are carrying a male fetus [64, 65]

3 2014 was the most recent database year available.

4 Health journals included the top five SSCI database journals (American Journal of Public Health, *Health Affairs*, *Social Science & Medicine*, *Epidemiology*, and *Medical Care*) and the top five SCIE database journals (*New England Journal of Medicine*, *Lancet*, *Journal of the American Medical Association*, *British Medical Journal*, and *Cochrane Database of Systematic Reviews*). Sex/gender focused health journals included the top ten relevant journals from both databases (*Journal of Women’s Health*, *Women’s Health Issues*, *Journal of Midwifery & Women’s Health*, *Gender Medicine: The Journal for the Study of Sex & Gender Differences* (formerly *Women’s Health & Gender-Based Medicine*), *BMC Women’s Health*, *Women & Health*, *American Journal of Men’s Health*, *Health Care for Women International*, *Women & Birth*, and *Journal of Men’s Health* (formerly known as the *Journal of Men’s Health & Gender*).

5 Key funding agencies included 45 agencies across 36 countries: the Australian Research Council (ARC), National Health and Medical Research Council (NHMRC) (Australia); Austrian Research Promotion Agency (FFG), Austrian Science Fund (FWF) (Austria); Agency for Innovation by Science and Technology—in Flanders (IWT), The Research Foundation - Flanders (FWO), Fund for Scientific Research (FRS-FNRS)—FWO (Belgium); National Science Fund (NSF) (Bulgaria); Canadian Institutes of Health Research (CIHR) (Canada); Croation Science Foundation (HRZZ) (Croatia); Cyprus Research Promotion Foundation (CRPF) (Cyprus); Czech Science Foundation (GACR) (Czech Republic); The Danish Council for Independent Research (DFF) (Denmark); Estonian Research Council (ETAG) (Estonia); The Academy of Finland (AKA) (Finland); French Centre National de la Recherche Scientifique (CNRS), French National Research Agency (ANR) (France); German Research Foundation (DFG); The National Council for Research and Technology (NCRT) (Greece); Hungarian Scientific Research Fund (OTKA) (Hungary); The Icelandic Centre for Research (RANNIS) (Iceland); Irish Research Council (IRC), Science Foundation Ireland (SFI) (Ireland); Israel Science Foundation (ISF) (Israel); Ministry of Education, Universities and Research (MIUR) (Italy); Latvian Council of Science (LZP) (Latvia); Research Council of Lithuania (LMT) (Lithuania); National Research Fund (FNR) (Luxembourg); Malta Council for Science and Technology (MCST) (Malta); Netherlands Organization for Scientific Research (NWO), Netherlands Organization for Health Research and Development (ZonMw) (Netherlands); Research Council of Norway (RCN) (Norway); National Centre for Research & Development (NCBIR), National Science Centre (NCN) (Poland); Foundation for Science and Technology (FCT) (Portugal); Executive Agency for Higher Education and Research Funding (UEFISCDI) (Romania); Slovak Research and Development Agency (SRDA) (Slovakia); Slovenian Research Agency (ARRS) (Slovenia); Secretary of State of Research, Development and Innovation (SEIDI) (Spain); Swedish Research Council (SRC) (Sweden); Swiss National Science Foundation (SNSF) (Switzerland); Scientific and Technological Research Council of Turkey (TÜBI˙TAK) (Turkey); Research Councils UK (RCUK), Medical Research Council (MRC) (UK); and US National Institutes of Health (NIH) (US).
